# Upregulation of synaptotagmin IV inhibits transmitter release in PC12 cells with targeted synaptotagmin I knockdown

**DOI:** 10.1186/1471-2202-11-104

**Published:** 2010-08-24

**Authors:** Johnnie M Moore-Dotson, Jason B Papke, Amy B Harkins

**Affiliations:** 1 Department of Pharmacological and Physiological Science, Saint Louis University School of Medicine, St. Louis, MO 63104, USA

## Abstract

**Background:**

The function of synaptotagmins (syt) in Ca^2+^-dependent transmitter release has been attributed primarily to Ca^2+^-dependent isoforms such as syt I. Recently, syt IV, an inducible Ca^2+^-independent isoform has been implicated in transmitter release. We postulated that the effects of syt IV on transmitter release are dependent on the expression of syt I.

**Results:**

To test this, we increased syt IV expression in PC12 cells by either upregulation with forskolin treatment or overexpression with transfection. Two separately generated stable PC12 cell lines with syt I expression abolished by RNAi targeting were used and compared to control cells. We measured catecholamine release from single vesicles by amperometry and neuropeptide Y release from populations of cells by an immunoassay. In syt I targeted cells with forskolin-induced syt IV upregulation, amperometry measurements showed a reduction in the number of release events and the total amount of transmitter molecules released per cell. In cells with syt IV overexpressed, similar amperometry results were obtained, except that the rate of expansion for full fusion was slowed. Neuropeptide Y (NPY) release from syt I knockdown cells was decreased, and overexpression of syt IV did not rescue this effect.

**Conclusions:**

These data support an inhibitory effect of syt IV on release of vesicles and their transmitter content. The effect became more pronounced when syt I expression was abolished.

## Background

Synaptic transmission is a highly regulated process that relies on calcium (Ca^2+^) to trigger the release of transmitters from membrane bound vesicles. To accomplish this fusion of vesicle and plasma membranes, the SNARE (soluble NSF receptor) complex of proteins is required as well as a Ca^2+ ^sensor that can bind Ca^2+ ^[1-4]. Synaptotagmin (syt) I is well established as a main Ca^2+ ^sensor for rapid and regulated vesicle release from neurons and neurosecretory cells [3,4]. It binds Ca^2+^, interacts with phospholipid membranes, and triggers the SNARE complex proteins to allow vesicle fusion and subsequent transmitter release [3,4].

Synaptotagmins are a conserved family of proteins comprised of at least 17 different isoforms (see Pubmed nucleotide ID# NM 138849, [5]). A distinguishing feature of the syt isoforms is the tandem cytoplasmic C2 domains (C2A, C2B) that, for some isoforms, confer the phospholipid and Ca^2+^-binding ability to the specific isoforms [6-8]. Although syt isoforms share similar C2 domain structures, they differ in their ability to bind Ca^2+ ^by the C2 domains. The Ca^2+^-dependent syt isoforms bind to phospholipids as a function of Ca^2+^-binding to the C2 domains and include syt I [7,9,10]. Conversely, syt IV is a unique syt isoform that has similar C2 domain structure, but does not bind Ca^2+ ^in the C2A domain due to an amino acid substitution that prevents Ca^2+^-binding and presumably prevents the Ca^2+^-dependent functions attributed to the C2A domain [6,7,10,11]. Unlike the C2A domain of syt IV, the C2B domain is thought to retain the ability to bind Ca^2+^, and this binding of Ca^2+ ^to the C2B domains of other syt isoforms has been shown to be essential for transmitter release [12-14]. Therefore, controversy remains as to whether syt IV can support Ca^2+^-dependent vesicle fusion and subsequent transmitter release.

An intriguing feature of syt IV is that it is an immediate early gene whose expression is induced *in vivo *following treatment with kainic acid to induce seizures [15]. In contrast, gene expression of syt I is either unchanged or decreased with kainic acid treatment [15-17]. Forskolin and depolarization with high K^+ ^leads to increased syt IV expression in the neuroendocrine rat pheochromocytoma (PC12) cells, mimicking the effects of kainic acid [15,16]. In mice, lack of syt IV has been associated with memory and performance deficits [18], as well as depression-like behaviors [19]. These behavioral effects in animals indicate that syt IV may play a role in synaptic transmission, transmitter and/or vesicle release processes.

Studies performed in PC12 cells and *Drosophila *have shown that overexpression of syt IV decreases transmitter release by the formation of hetero-oligomers with syt I [20-22]. More recently, forskolin-induced syt IV expression has been shown to decrease the fusion pore stability that leads to a more readily closed fusion pore, and thus, an increase in the frequency of 'kiss and run' events [23,24]. In syt IV knockout mice, vesicle release from the posterior pituitary nerve terminals [25] and hippocampal neurons [26] was enhanced compared to wild type mice. Taken together, these results have contributed to the hypothesis that syt IV acts to prevent neurotransmitter release during periods of high stimulation. However, in a separate study performed in mouse hippocampal neurons, there were no measurable effects of syt IV upregulation on fast synaptic transmission, fusion modes or kinetics [27].

In *Drosophila*, syt IV has been shown to substitute for syt I to mediate transmitter release [14], or in pituitary gonadotropes, to be necessary for large dense core vesicle (LDCV) release when syt I was not effective [28]. Furthermore, in glial cells, syt IV was shown to be essential for Ca^2+^-dependent release of glutamate [14,29]. In most cases, experiments done to investigate the involvement of syt IV on transmitter release were performed in secretory cells that also expressed the primary Ca^2+^-sensor, syt I. In fact, because syt IV has been shown to co-localize with syt I and reported to form hetero-oligomers [20-23,30-32], it is possible that the function of syt IV in regulated release of vesicles is dependent upon whether syt I is also expressed.

We hypothesize that syt IV modulates Ca^2+^-regulated transmitter release and that the function of syt IV is dependent upon the presence of syt I. To test this hypothesis, we used PC12 cell lines established previously in our laboratory. These cell lines have stable incorporation of plasmid-based RNAi to specifically eliminate expression of syt I [33]. We have already shown that abolished syt I expression reduced catecholamine (CA) release by ~50% from single cells [33]. Furthermore, we showed that evoked release of neuropeptide Y (NPY) from LDCVs was reduced in the absence of syt I, establishing a role for syt I in differentially regulating transmitter release from LDCVs compared to small vesicles in PC12 cells [34]. In the current study, we show that syt IV upregulation does not affect CA release or NPY release when syt I is expressed. However, upregulation of syt IV in the absence of syt I expression reduces release of vesicles and their transmitter content compared to release from cells that have endogenous expression of syt IV with targeted knockdown of syt I. Together, these data show that neither upregulation nor overexpression of syt IV substitutes for syt I.

## Results

### Expression of syt IV is upregulated by forskolin in syt I knockdown cells

In a previous study, we established a PC12 cell line that continuously expresses a short hairpin (sh)RNA designed to silence expression of syt I, that we refer to as shRNA-syt I (or shRNA-syt Ia if more than one stable line was used) [33]. In that study, we showed that expression of Ca^2+^-dependent syt isoforms II, III, VII and IX, and the SNARE proteins were unaltered by the lack of syt I expression in the shRNA-syt I cells. However, we did not characterize the expression levels of syt IV. Previously, other researchers had shown that syt IV expression is rapidly increased in PC12 cells by treatment with forskolin [15,35] which is thought to act on adenylyl cyclase, a potent activator of cAMP production that results in activation of multiple signal transduction pathways including CREB-mediated transcription [36,37]. It is postulated that forskolin-induced syt IV expression is achieved by CREB-mediated transcription [16]. Therefore, we treated control PC12 and syt I knockdown cells with 50 μM forskolin, and used immunodetection methods to determine whether upregulation of syt IV expression was increased when cells have abolished syt I expression [15,35].

**Figure 1:**
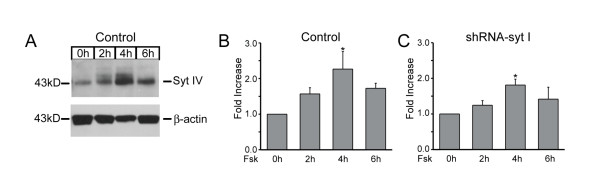
**Synaptotagmin IV (syt IV) expression is increased by forskolin**. Control and syt I knockdown cells were treated with 50 μM forskolin (fsk) for 0, 2, 4 and 6 hours. *(A) *Immunoblot analysis shows that syt IV expression is upregulated after continuous fsk treatment for 4 hours in control PC12 cells. β-actin expression remained constant, and was used as a loading control. All lanes were loaded at 20 μg/lane. Syt IV expression was normalized to arbitrary densitometry units of β-actin measured in control PC12 cells *(B)*, n = 7, and in shRNA-syt I knockdown cells *(C)*, n = 4. Syt IV expressed peaked at 4 hours in both cell types (*p < 0.05).

Representative immunoblots of lysates prepared from control PC12 and syt I knockdown cells show that syt IV expression was induced within 2 hours of continuous forskolin treatment. Figure 1A shows an example immunoblot of syt IV upregulation in response to forskolin treatment measured from control PC12 cells normalized to β-actin. Similar results were obtained in shRNA-syt I cells. Intracellular cAMP was measured with an immunoassay, and increased approximately 200 to 300-fold in each cell type as expected. The plots in Figures 1B and C show the average fold increase in syt IV expression levels measured in forskolin-treated control and shRNA-syt I knockdown cells compared to untreated cells. Densitometry analysis shows that syt IV expression was significantly increased ~2.3-fold (n = 7) and peaked after 4 hours of treatment with forskolin in control PC12 cells compared to untreated cells (Figure 1B). Likewise, syt IV expression in syt I knockdown cells increased ~2.0-fold (n = 4) and peaked after 4 hours of forskolin treatment (Figure 1C). These results are similar to previous reports that described an increase in syt IV mRNA and protein expression within 4 hours of treatment with forskolin [15,35]. We found similar results in control transfected (CT, n = 4) cells that were stably transfected with an empty plasmid that lacks a targeting insert and does not express any shRNAs. Furthermore, to be certain that upregulating syt IV does not affect expression of syt I, we determined that syt I expression was unaffected by forskolin treatment and remained constant over time in forskolin-treated control cells.

### Upregulation of syt IV inhibits CA release in syt I knockdown cells

**Figure 2:**
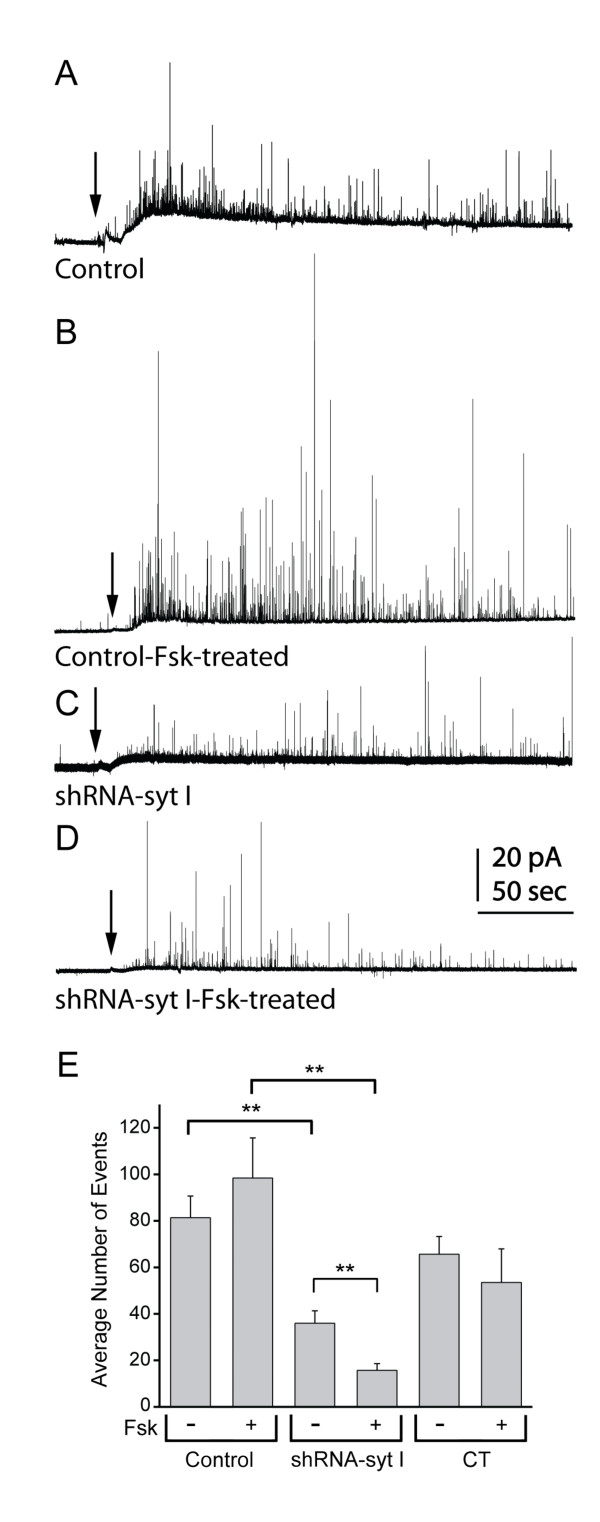
**Syt IV expression in syt I knockdown cells reduced evoked secretion**. Representative amperometry recordings are shown from an untreated control cell *(A)*, forskolin-treated control cell *(B)*, untreated shRNA-syt I knockdown cell *(C)*, and a forskolin-treated shRNA-syt I knockdown cell *(D)*. The arrows depict where the continuously perfused solution was changed from HBSS to 50 mM K^+ ^stimulating solution. *(E) *The average number of events per cell group is shown in the plot for forskolin-treated control (n = 19), shRNA-syt I knockdown (n = 16), and control transfected, CT (n = 12) cells, compared to untreated control (n = 24), shRNA-syt I knockdown (n = 24) and CT (n = 15) cells (**p < 0.01).

**Figure 3:**
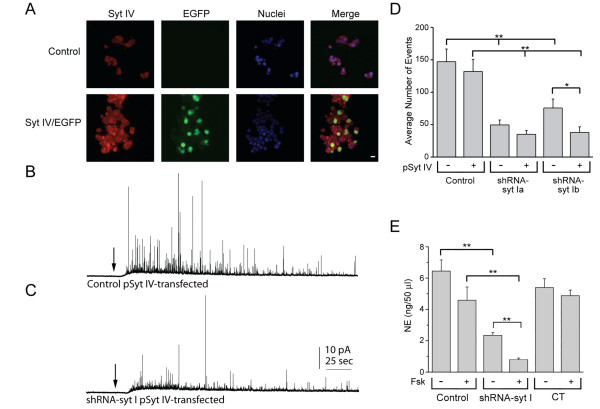
**Overexpression of syt IV has similar effects on secretion from the cells as upregulation of syt IV**. *(A) *Confocal images of cells are shown stained with fluorescently labeled antibodies to syt IV (red), EGFP (green), and nuclei (blue). The merge of all three images is shown on the right. The top group of images shows endogenous staining for syt IV in control cells that have not been transfected with the plasmids that express syt IV and EGFP. The lower group of images shows cells cotransfected with syt IV/EGFP plasmids in a 10:1 ratio. The images are representative of 3 independent experiments (scale bar = 10 μm). *(B) *Amperometry recordings are shown in a representative control cell transfected with the plasmid that expressed syt IV (pSyt IV) *(C) *and in an shRNA-syt I knockdown cell transfected with the pSyt IV *(B)*. The arrows depict where the continuously perfused solution was changed from HBSS to 50 mM K^+ ^stimulating solution. *(D) *The average number of events is plotted for control cells (n = 16) and two independent stable syt I knockdown cell lines (syt Ia, n = 16, and syt Ib, n = 20) compared to cells cotransfected with human syt IV/EGFP (control, n = 13 cells; syt Ia, n = 17 cells; and syt Ib, n = 17 cells; *p < 0.05 and **p < 0.01). *(E) *Basal and stimulated norepinephrine (NE) release were analyzed from 9-21 independent experiments. NE release is expressed as total NE (ng) per 50 μl sample (**p < 0.01).

To test the hypothesis that syt I and syt IV are functionally co-dependent in regulating exocytosis of transmitter from vesicles, we used carbon-fiber amperometry to detect the release of individual vesicles and secretion of transmitter. We recorded amperometric release events from control cells, shRNA-syt I knockdown cells, and control transfected cells, each of which were either untreated or treated with forskolin for 3-5 hours. Baseline release was measured during the first 20-30 sec of each recording while cells were continuously bathed in a Hank's Basal Salt Solution to establish the resting conditions. The solution was then changed to a stimulating solution of high K^+ ^(arrow in the amperometry traces, Figures 2A-D, and 3B and 3C), and continuously perfused for 4 min. Figure 2 shows representative examples of the recordings obtained from control PC12 cells compared to cells treated with forskolin for 3-5 hours (Figures 2A and 2B). Figures 2C and 2D show examples of amperometric recordings from syt I knockdown cells compared to syt I knockdown cells treated with forskolin. The syt I knockdown cell treated with forskolin (Figure 2D) exhibits reduced numbers of events compared to the control, forskolin-treated cell (Figure 2B).

The plot in Figure 2E shows the average number of events per cell measured during the 4 min stimulation period. There was no significant difference in the events measured in forskolin-treated control cells compared to untreated control cells. However, the average number of events measured in forskolin-treated shRNA-syt I cells was reduced by 84% compared to the number of events measured in forskolin-treated control PC12 cells (p < 0.01). Furthermore, the average number of events in forskolin-treated syt I knockdown cells was significantly reduced compared to the untreated syt I knockdown cells. Therefore, when syt IV is upregulated with forskolin, syt I functionally allows release of catecholamine-containing vesicles; whereas, when syt I is abolished by the shRNA targeting syt I, upregulation of syt IV causes an even greater reduction in release events.

### Stimulated influx of intracellular Ca^2+ ^is not reduced after forskolin treatment in syt I knockdown cells

In addition to activating CREB-mediated transcription, in some cells forskolin-induced cAMP production also leads to release of Ca^2+ ^from intracellular stores [38]. We were concerned that forskolin treatment could result in a secondary increase in intracellular Ca^2+ ^levels ([Ca^2+^]_i_) and induce transmitter release. To determine whether forskolin treatment altered levels of intracellular [Ca^2+^]_i _by release of Ca^2+ ^from intracellular stores or influx of extracellular Ca^2+^, we used fura-2 to image Ca^2+ ^and measured relative levels of resting and stimulated [Ca^2+^]_i_. Resting Ca^2+ ^levels in untreated and forskolin-treated control, syt I knockdown and CT cells were unaffected by forskolin-treatment (data not shown). With high K^+ ^stimulation, each group of cells responded with an expected ~4-fold increase in [Ca^2+^]_i_. Treatment with forskolin did not enhance or prevent influx of extracellular Ca^2+ ^in response to depolarization in any of the cell groups (data not shown). Thus, forskolin does not alter extracellular Ca^2+ ^entering the cell, nor does it induce or alter intracellular release of Ca^2+ ^from stores. This is in agreement with previous results that show that some clonal lines of PC12 cells do not have major intracellular Ca^2+ ^stores [39,40], or that some PC12 cells lack the receptors to activate release from stores [41].

### Overexpression of syt IV confirms that syt IV inhibits CA release in syt I knockdown cells

To determine that the reduced transmitter release was specific to syt IV upregulation and not due to other effects caused by forskolin-mediated cAMP production, we transiently co-transfected the cells with two plasmids, one that expresses the human isoform of syt IV and one that expresses enhanced green fluorescent protein (EGFP) in a 10:1 μg ratio, respectively. Transfections were performed in control PC12 cells and two different, independent, stable syt I knockdown cell lines (shRNA-syt Ia and Ib) that each had abolished expression of syt I. To determine that the exogenously expressed syt IV protein was expressed in EGFP-expressing PC12 cells, we used immunocytochemistry with an anti-syt IV antibody that detects both human and rat syt IV, and an antibody that detects EGFP. Figure 3A shows untransfected (top) and co-transfected (bottom) PC12 cells stained for syt IV (red), EGFP (green), and nuclei (blue). In the top images, the syt IV antibody shows that syt IV is endogenously expressed at low levels in the control PC12 cells. From an image analysis using ImageJ software, the percentage of fluorescing pixels/cell was measured from the red channel depicting syt IV expression and from the blue channel depicting the nuclei stain. The ratio of the pixel percentages was compared between the red and blue channels. In the lower images, overexpressed syt IV is expressed at greater levels (~15-fold) than endogenously expressed syt IV. A merge of the red and green images shows that co-transfected cells stained for both syt IV and EGFP. From three independent experiments, we determined that nearly 100% of the EGFP-expressing cells also stained for overexpressed syt IV. Therefore, we were confident that when we recorded from EGFP-expressing cells that those chosen cells also overexpressed the plasmid expressing human syt IV.

We used amperometry to measure individual release events from the control and syt I knockdown cells that were co-transfected with syt IV and EGFP plasmids. Figure 3B shows an example amperometry recording from a control cell (that endogenously expresses syt I) and that has overexpressed syt IV. Figure 3C shows an example recording of a syt I knockdown cell that has overexpressed syt IV. After ~20 sec of baseline recording, the solution was changed to a high K^+ ^stimulating solution for 4 min (arrows in Figures 3B and 3C). The plot in Figure 3D shows that the average number of events per cell was reduced (p < 0.01) in the two independent stable syt I knockdown cell lines (shRNA-syt Ia and Ib, no syt IV expressed) compared to the non-transfected control cells, as expected for cells that lack syt I. There was also a reduction (p < 0.01) in the number of events per cell between the control cells and syt I knockdown cells (Ia and Ib) when syt IV was overexpressed. Although there was no difference in the average number of events in control cells that express endogenous levels of syt I when transfected with pSyt IV compared to non-transfected cells (two leftmost bars), one of the two syt I knockdown cell lines (Ib) showed a significant decrease (p < 0.05) in the average number of events when syt IV was overexpressed compared to non-transfected syt Ib knockdown cells. The total number of events was reduced for the syt I knockdown cells compared to control cells. The total number of events was reduced further when syt IV was overexpressed compared to the non-transfected cells. These numbers of events follow a similar trend as seen in the syt I knockdown cells that had syt IV upregulated by forskolin.

To confirm that the reduced number of release events detected from individual cells was characteristic of the population of forskolin-treated syt I knockdown cells, high performance liquid chromatography-electrochemical detection (HPLC-EC) was used to measure norepinephrine (NE) release from populations of cells. Figure 3E shows the amount of NE release detected from each group of cells after 5 min of stimulation with high K^+^. There were no significant differences between control and CT cells with or without forskolin treatment. Similar to the single vesicle release measurements detected with amperometry, stimulated NE release was significantly reduced (p < 0.01) in untreated syt I knockdown cells compared to untreated control cells, as well as in forskolin-treated syt I knockdown cells compared to forskolin-treated control cells. Stimulated release of NE was decreased by half in forskolin-treated syt I knockdown cells compared to untreated syt I knockdown cells (p < 0.05).

### Syt IV does not alter amplitude or quantal content of vesicle release

**Figure 4:**
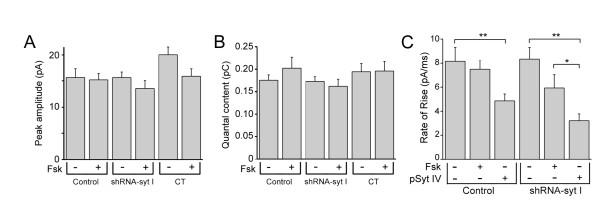
**The rate of the fusion pore opening is slowed in syt I knockdown cells that overexpress syt IV, but neither the amplitude nor quantal content of the individual release events are altered following syt IV upregulation**. The 'double' means of the amplitude *(A) *and quantal content *(B) *values are shown in bar histograms for forskolin-treated and untreated control, shRNA-syt I knockdown, and CT cells. *(C) *The mean rate of rise (pA/ms) for each group of cells is plotted for the control and shRNA-syt I knockdown cells with control, endogenous levels of syt IV expressed, upregulated syt IV expression by forskolin treatment, or overexpression of syt IV by transfection with pSyt IV (*p < 0.05, **p < 0.01).

Individual amperometric spike events can be analyzed on expanded time scales to evaluate such parameters as spike amplitude and quantal content that are correlated to the amount of neurotransmitter molecules contained within the vesicles [42]. Previously, others have determined that quantal content is correlated to the relative diameter of the vesicle, with small events corresponding to small vesicles, and larger events corresponding to LDCVs [43]. Furthermore, analysis of the spike kinetics describe the fusion pore expansion by the rate of rise, and the half-width of the spike corresponds to the diffusion of transmitter from the vesicle as the vesicle fuses with the plasma membrane [44]. Quantitative analysis of the amperometric spike shows that the mean peak amplitude was similar in untreated and forskolin-treated control cells, syt I knockdown cells and CT cells (Figure 4A). The integrated area under the spike events is calculated as the charge or quantal content per vesicle shown in Figure 4B. There were no significant differences regardless of forskolin-treatment in control and syt I knockdown cells in the quantal content per vesicle released. This indicates that the amount of transmitter released per event was not different between the cells that had syt IV upregulated or abolished syt I expression.

**Figure 5:**
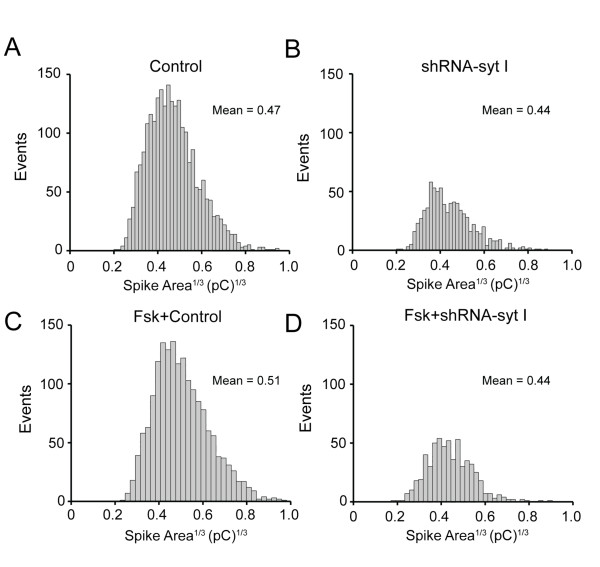
**The overall number of events are reduced by syt IV upregulated in syt I targeted cells, but without any loss of the small or large diameter vesicle populations**. Cumulative distribution histograms of the cubed root of the quantal content (pC)^1/3 ^are shown for untreated and forskolin-treated control *(A, C) *and shRNA-syt I knockdown *(B, D) *cells. The mean of each histogram is displayed on the individual graph generated with 48 bins.

A Gaussian distribution of measured quantal content will depict the range of vesicle diameters released from cells. Integrated charge over time (*Q*) is proportional to vesicle volume assuming that transmitter concentration is the same for all vesicles and that release of vesicle content results in complete emptying. Under these assumptions, the cubed root of the quantal charge (Q^1/3^) is directly proportional to vesicle diameter [43,45]. To determine whether a specific population of vesicles had failed to be released from cells that had increased syt IV expression, a cumulative distribution histogram of the cubed root of quantal content (Q^1/3^) was plotted versus the number of events for all of the control and syt I knockdown cells (Figure 5A-D). In each histogram (where bin size was set to 48), the data for the untreated control and forskolin-treated control cells are best described by a single Gaussian. The means of the histograms are shown on each plot, and are similar to one another, ranging from 0.44 pC^1/3 ^to 0.51 pC^1/3^. Each of the syt I knockdown cell groups, either with or without forskolin treatment to upregulate syt IV, were similar to control cells in their distribution of vesicle population. However, the total number of events was largely reduced in the syt I knockdown cells which affects the amplitude of the distribution histogram. Because the means and distributions exhibit similar cubed roots of quantal content (Q^1/3^), there was no apparent loss of one population of vesicles, only an apparent reduction in total number of vesicles released from syt I knockdown cells.

Analysis of the amperometry events measured from each type of cell with overexpressed syt IV had similar peak amplitudes and quantal contents as the cells with upregulated syt IV using forskolin. Additionally, the cubed root of the quantal content and Gaussian histograms gave similar ranges of means, and no apparent loss of vesicle population. Therefore, in PC12 cells that express endogenous syt I or cells stably transfected with shRNA to target syt I knockdown, there is no difference between averaged amplitudes or quantal content of packaged transmitter when syt IV is upregulated or overexpressed, nor is there any specific loss of one population of vesicles for release compared to another, such as small clear vesicles compared to LDCVs.

### Overexpression of syt IV alters the fusion kinetics

We analyzed each of the release events measured from the various groups of cells to determine whether the properties of the fusion pore expansion and transmitter diffusion were affected when syt IV expression was upregulated by forskolin treatment, or overexpressed by transfection, compared to control cells. There were no differences in the half-width of the spikes for either the upregulated syt IV or overexpressed syt IV. Figure 4C shows the rate of rise (pA/ms) was significantly slowed in both the control cells and the syt I knockdown cells when syt IV was overexpressed, but not when syt IV was upregulated with forskolin treatment. The rising phase exhibited the same slowed kinetics. Therefore, overexpression of syt IV slowed the rate of fusion pore expansion, but did not affect the rate of transmitter diffusion from the vesicle (half-width).

### Syt IV reduced the number of transmitter molecules released from syt I knockdown cells

**Figure 6:**
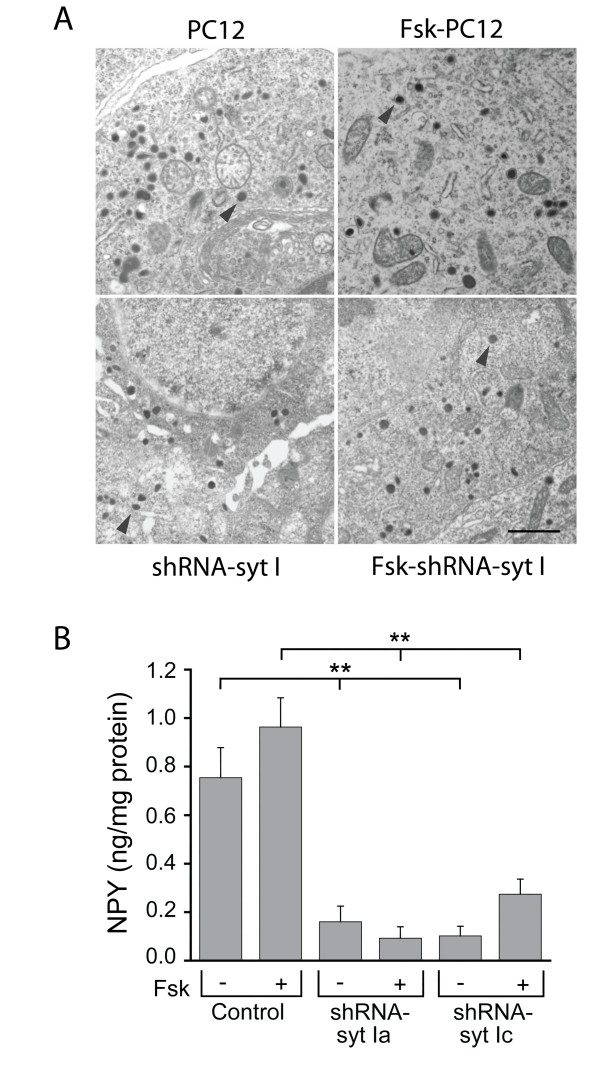
**LDCVs are synthesized and NPY release remains reduced in shRNA-syt I knockdown cells when syt IV is upregulated by forskolin treatment**. (*A*) Large dense core vesicles are apparent in both control and fsk-treated cells. Electron micrographs reveal that in the control PC12 cells (top panels) and in the syt I knockdown cells (lower panels), both untreated (left side) and fsk-treated (right side) have large dense core vesicles (example shown in each panel by arrowhead). Scale bar = 500 nm. (*B*) The average stimulated NPY release from 9-12 independent experiments is plotted. Untreated and forskolin-treated control cells, and two independent syt I knockdown cell lines (shRNA-syt Ia and shRNA-syt Ic) were stimulated for 15 min with high K^+ ^to evoke NPY release (**p < 0.01).

The Faraday equation, *N *= *Q*_*tot*_/*ne*, calculates the number of oxidized CA molecules per cell (*N*) released, where *Q_tot _*is total charge per cell, *n *is two electrons per oxidized molecule of CA, and *e *is the elemental charge (1.602 × 10^-19 ^C). For each cell, we calculated the total charge per cell (*Q_tot_*) by multiplying the average charge measured per vesicle (*Q*) by the total number of events per cell. Tables 1 and 2 show the summary of the averaged released number of CA molecules/cell for each group. In Table 1, the number of molecules released from untreated syt I knockdown cells were reduced compared to the untreated control cells (p < 0.01), as were the number of molecules released from forskolin-treated syt I knockdown cells compared to forskolin-treated control cells (p < 0.01). Similar results are shown in Table 2 that compares the effects of overexpression of syt IV in the syt I knockdown cells to control cells. To be certain that the reduction in number of molecules in the syt I knockdown cells were not due to a failure of the cells to synthesize LDCVs, we confirmed that each of the groups of cells contained LDCVs with electron microscopy (Figure 6A). However, without three-dimensional reconstructions of cells, it is not clear if there is a reduction in the number of synthesized vesicles, or if LDCVs have a smaller volume reflecting a possible reduced content of stored CA. Even so, these data together indicate that syt IV does not substitute for syt I to support evoked release, but instead, upregulation of syt IV expression further inhibits release of vesicle contents.

**Table 1:** CA molecules released from forskolin-treated control and shRNA-syt I knockdown cells.

	**Average Number of CA Molecules/cell (× 10**^**6**^**) ± SEM (× 10**^**6**^**)**	Number of Cells
Control	47.5 ± 7.7	24
Fsk-Control	51.5 ± 8.3	19
shRNA-syt I	17.7 ± 3.1*	24
Fsk-shRNA-syt I	8.8 ± 1.9^+,±^	16

The Faraday equation was used to calculate the average number of CA molecules per cell from the total charge per cell [44](*p < 0.01 compared to control cells; ^+^p < 0.01 compared to fsk-control cells; and ^±^p < 0.05 compared to shRNA-syt I cells).

**Table 2:** CA molecules released from control and shRNA-syt I knockdown cells transfected with pSyt IV.

	**Average Number of CA Molecules/cell (× 10**^**6**^**) ± SEM (× 10**^**6**^**)**	Number of Cells
Control	56.7 ± 7.2	16
pSyt IV-Control	64.3 ± 13.8	13
shRNA-syt Ia	16.0 ± 2.9**	16
pSyt IV-shRNA-syt Ia	11.1 ± 2.3^+^	17
shRNA-syt Ib	33.2 ± 7.8*	20
pSyt IV-shRNA-syt Ib	11.0 ± 2.4^+,±^	17

The Faraday equation was used to calculate the average number of CA molecules per cell from the total charge per cell [44] (*p < 0.05 and **p < 0.01 compared to control cells; ^+^p < 0.01 compared to pSyt IV control cells; and ^±^p < 0.05 compared to shRNA-syt Ib cells).

### Stimulated NPY release is not rescued by syt IV

The results described above summarize the effect of forskolin-induced syt IV expression on regulated CA release in the presence and absence of syt I. CAs are reported to be localized to both small vesicles and LDCVs [46-48]. Previous reports show that syt IV is localized to LDCVs and regulates the fusion mechanism for LDCVs in syt I-expressing PC12 cells [23,24]. The "core" of LDCVs is composed of many secretory peptides including NPY [49,50]. Therefore, we tested whether syt IV mediates the release of NPY from LDCVs, and whether this is dependent upon the presence of syt I.

We used an immunoassay to measure stimulated NPY release from control and syt I knockdown cells treated with forskolin and compared to untreated cells. Figure 6B shows that NPY release was greatly reduced in two independent syt I knockdown cell lines compared to control cells. Both shRNA-syt Ia and Ic stable cell lines exhibit reduced NPY release normalized to total protein compared to control cells (p < 0.01). Similarly, in the same cells that had upregulated syt IV expression following forskolin treatment, release of NPY remained significantly reduced compared to control cells treated with forskolin (p < 0.01). Reduced release of NPY was not rescued by forskolin-upregulated syt IV expression. Thus, syt IV does not act redundantly for syt I to restore NPY release in syt I knockdown cells.

## Discussion

In the current experiments, we have studied the role that syt IV plays in regulating release of CAs and NPY from vesicles without interference from the primary syt isoform, syt I. Previously, we showed that syt I plays a differential role in regulating release of these two transmitters [33,34]. In this study, we show that in cells that express syt I, neither upregulated syt IV nor overexpressed syt IV affects the number of CA release events measured from single cells (Figures 2E and 3D, respectively), nor does syt IV affect CA release measured from populations of cells by HPLC (Figure 3E). Similarly, in cells that expressed syt I, there were no effects of syt IV upregulation on NPY release (Figure 6B).

Conversely, in cells that had syt I expression abolished, the number of CA release events was reduced compared to control cells that endogenously expressed both syt I and IV (Figures 2E and 3D). In the syt I knockdown cells, increased syt IV expression resulted in reduced CA release events. In a recent study by Arthur et al. (2010), they speculated that syt IV knockout mice have vesicles that are more fusogenic, which would support our finding that increased syt IV expression results in less fusogenic vesicles. NPY release was also reduced in these cells, but not reduced any more than what was observed in cells that lacked syt I with endogenous syt IV levels (Figure 6B). Previously, there was speculation that syt IV mediates neuropeptide secretion from LDCVs [31], however, we did not observe any rescue of NPY release in cells that lacked syt I and expressed syt IV (Figure 6B). Recently, in a study measuring exocytosis of oxytocin and vasopressin, peptides secreted from LDCVs from the posterior pituitary, syt IV was shown to affect the Ca^2+ ^dependency for exocytosis [25]. In that study, syt IV knockout mice responded to low levels of Ca^2+ ^entry with greater exocytosis of vesicles, and to higher levels of Ca^2+ ^with less exocytosis of vesicles [25]. Therefore, in PC12 cells, syt IV plays an inhibitory role in vesicle release that is revealed when syt I is not expressed. Together, these data indicate that syt IV does not substitute for syt I to support Ca^2+^-dependent release of transmitter from vesicles.

The average distribution of quantal content was measured for each of the upregulated syt IV and syt I knockdown cells, and compared to control cells. From the cubed root analysis of the average charge measured per vesicle (*Q*) there was no apparent loss of one population of vesicles over another based on means of the distribution histograms in Figure 5, only an apparent reduction in total number of vesicles released from syt I knockdown cells. Average amplitude and charge per vesicle are unaffected (Figures 4A and 4B, respectively). The number of CA molecules released was not significantly different when syt IV was upregulated or overexpressed in syt I expressing cells (Tables 1 and 2). However, there was a reduction in number of CA molecules released when control cells were compared to syt I knockdown cells with endogenous expression of syt IV (Tables 1 and 2). A similar reduction of CA molecules released was observed for the control syt I knockdown cells when syt IV expression was either upregulated or overexpressed (Tables 1 and 2). Our results agree with two previous studies that each used overexpression of syt IV in PC12 cells in the presence of syt I: a decreased number of transmitter molecules was released [23], and many fewer vesicles were released than from control cells without increasing extracellular stimulation conditions [51].

Many possibilities could explain the reduced number of CA molecules released from cells that have syt I expression abolished and syt IV expression increased. The first possibility is that forskolin treatment causes a reduction of intracellular Ca^2+ ^influx; however, we report here that forskolin does not alter the influx of intracellular Ca^2+ ^in any of the cell groups (data not shown). A second possibility is that release of fewer CA molecules is due to a reduction in the number of vesicles synthesized. In fact, in a recent study from syt IV knockout mice, synaptic vesicles were reduced in numbers synthesized, and in their targeting to proper release sites [52]. We compared electron micrographs from control and syt I knockdown cells that had endogenous syt IV levels expressed or syt IV levels upregulated by forskolin, and each cell group had LDCVs readily apparent. Therefore, LDCVs were synthesized in each of the cells; however, without serial reconstruction of the cells, we cannot quantify or properly localize the vesicles to address a possible reduction in total number of vesicles synthesized. Furthermore, syt IV may be inhibiting Ca^2+^-triggered vesicle fusion mediated via the SNARE/phospholipids, an effect that becomes more pronounced when syt I is not expressed, thus revealing a role for syt IV that is masked when syt I is expressed. Interestingly, in a study using *Drosophila *neuromuscular junction, evoked release could be inhibited by increasing the ratio of syt IV to syt I expression [22].

We show that kinetic analysis of amperometric spikes measured from both control and syt I knockdown cells that had overexpressed syt IV exhibited a reduced rate of rise of the spike, indicating that expansion of the fusion pore required a longer time to open to release the transmitter contents compared to control cells (Figure 4C). Fusion kinetics were unaffected in either syt I knockdown cells or control cells that had upregulated syt IV expression. No other kinetic changes were observed in any of the cell types with or without syt I targeted or in cells with increased syt IV expression. One possible explanation is that endogenous levels of syt IV expression are relatively low until increased by either forskolin treatment or overexpression. By overexpressing syt IV, we have bypassed the regulatory machinery of syt IV expression and syt IV is constantly expressed. Overexpression likely leads to unregulated and possibly greater expression of syt IV than by forskolin treatment, although because PC12 cells have low transfection efficiency, it is difficult to quantify the levels of overexpression of syt IV protein compared to upregulation by forskolin using immunoblot analysis. In a study in syt I-expressing hippocampal neurons that had overexpressed syt IV, no changes in fusion kinetics were observed [27], and in syt I-expressing PC12 cells that had syt IV overexpressed, decreased fusion pore stability was observed [23]. In our study, we have used the human syt IV rather than rat syt IV for the overexpression studies. Even though the sequence is mostly conserved, the human isoform may have somewhat different Ca^2+ ^binding properties than the rat isoform, which to our knowledge, has not been directly measured. However, differences in Ca^2+ ^binding affinities have been reported in *Drosophila*, whose syt IV isoform has been shown to have a higher affinity for Ca^2+ ^than rat syt IV [53]. Unlike *Drosophila *syt IV, rat syt IV does not participate in Ca^2+^-dependent binding of phospholipids [53,54]. Both homologues can form hetero-oligomers with other syt isoforms [20,22]. Additionally, syt IV has been shown to establish linear Ca^2+ ^dependence of vesicle release from mouse auditory ribbon synapses [55]. These results implicate syt IV as having a modulatory effect on vesicle release, that may be dependent upon an expressed ratio of syt I and syt IV in different vesicles, and at different synapses, an idea that was confirmed in a *Drosophila *study [22].

Because PC12 cells normally express low levels of endogenous syt IV, syt I may dominate the phenotype and therefore the function of syt IV by having a higher affinity for Ca^2+ ^and therefore outcompeting endogenous levels of syt IV for Ca^2+^. Alternatively, because it has been shown that syt I and IV colocalize at the same vesicles [23], by knocking down expression of syt I, another mechanism may become activated that compensates for syt I in the release machinery which is the target for syt IV. This observation would then become more apparent with an increase of syt IV expression in the absence of syt I.

## Conclusions

This study shows that syt IV inhibits release of transmitters from PC12 cells that lack the primary Ca^2+ ^sensor, syt I. We show that in syt I knockdown cells, CA and NPY release is reduced compared to control cells, and that regardless of how syt IV expression is increased, syt IV does not substitute to rescue the release. With the development of the syt IV knockout mouse, others have begun to determine how syt IV may function as a modulator of transmitter release. However, more studies are needed to determine the exact mechanism of action of a syt isoform that is Ca^2+^-independent, but that in some cases displays Ca^2+^-dependent activities and promotes release, while in other cases, acts to inhibit Ca^2+^-dependent transmitter release.

## Methods

### Antibodies

The anti-synaptotagmin IV goat polyclonal antibody was obtained from Santa Cruz Biotechnology (Santa Cruz, CA). The anti-β-actin mouse monoclonal antibody was obtained from Developmental Studies Hybridoma Bank (University of Iowa, Iowa city, IA).

### Plasmids

A plasmid encoding human syt IV was constructed from the pBluescriptR that contained the human syt IV DNA construct (ATCC, Manassas, VA; GenBank ID: BC036538, BI548768) and pcDNA 3.1 Neo (+) plasmid. The plasmid was sequenced, and matched 100% the expected human syt IV sequence. The EGFP-N1 plasmid was a kind gift from Dr. William Green (University of Chicago).

### Cell culture

An early passage of rat PC12 cells was maintained according to standard methods [56,57]. Cells were passaged every 7 days and after 10 passages, a new vial of cells from frozen stocks was thawed. Stably transfected cells were established as previously described [33,34]. For experiments that required forskolin treated cells, cells were incubated with media containing 50 μM forskolin from 0 to 6 h as specified in the results. Prior to experimentation, cells were plated to the appropriate plates or coverslips, also coated with collagen.

### Immunoblot analysis

Immunoblot experiments were performed as previously described [33]. Briefly, cells were grown to confluency on a 100-mm tissue culture dish, removed from plates and lysed in buffer containing 20 mM Tris (pH = 7.5), 1% Triton-X 100, 10% glycerol, 2 mM EDTA, and protease inhibitor cocktail (Roche, Indianapolis, IN). Protein concentration was determined by Coomassie Plus protein assay reagent (Pierce, Rockford, IL) or by the Quant-iT protein assay with the Qubit fluorometric method (Invitrogen-Molecular Probes, Carlsbad, CA). Total cell lysates were electrophoresed on a 10% NuPAGE Bis-Tris pre-cast gel (Invitrogen, Carlsbad, CA) and transferred to a polyvinylidene difluoride membrane (PVDF) (Millipore, Billerica, MA). The membrane was blocked overnight with milk and incubated with primary antibody (1:1000 dilution), followed by a horseradish peroxidase-conjugated secondary antibody (1:50,000 dilution, Santa Cruz Biotechnology, Santa Cruz, CA). Immunoreactive bands were detected and visualized using ECL Advance reagent (GE Healthcare, Piscataway, NJ), exposed to X-ray film and developed. The blots were stripped and reprobed for β-actin. Quantitative analysis was done using Image J http://rsb.info.nih.gov/ij/ to determine the relative amount of protein expression. Each immunoblot is representative of 3 or more independent experiments.

### Transfection

Cells were transfected with 3 μg of transfection grade human syt IV plasmid DNA and EGFP-N1 plasmid DNA at a 10:1 μg ratio per 35 mm tissue culture plates using Lipofectamine 2000 (Invitrogen, Carlsbad, CA). The cells were incubated with the transfection mixture for 4 hours. The cells were used in experiments 48 hours after transfection.

### Immunocytochemistry

Cells were replated to collagen-treated Permonox chamber slides and fixed one day later with 2% paraformaldehyde. The cells were permeabilized with 0.1% Triton X-100, blocked with 2 mg/ml BSA, and incubated with a primary anti-syt IV goat polyclonal antibody (1:50 dilution). The cells were washed and incubated with a second primary anti-EGFP Alexa Fluor-conjugated rabbit polyclonal antibody (1:2,000; Invitrogen-Molecular Probes, Carlsbad, CA). This was followed by incubation with a secondary donkey-anti-goat Alexa Fluor-conjugated antibody (1:200; Invitrogen-Molecular Probes, Carlsbad, CA) to detect syt IV. Nuclei were stained with TO-PRO3 and the cells were mounted with Vectashield (Vector Laboratories, Burlingame, CA). Images were acquired using a scanning confocal microscope (Bio-Rad MRC 1024; Hercules, CA) with a 4× zoom on a 60× oil-immersion lens (NA 1.4).

### Amperometry

Cells were replated to plastic sterile coverslips 24-48 hours prior to experimentation and preloaded with 2 mM norepinephrine (Sigma, St. Louis) for 2-5 hours at 37°C. Amperometric electrodes were fabricated by threading a glass capillary tube with a 6 μm diameter carbon-fiber (Goodfellow, Huntingdon, England). The electrodes were pulled, sealed with epoxy, and stored at 95°C until use. The electrode was backfilled with 3 M KCl, clamped at +700 mV versus a Ag-AgCl ground with an NPI amplifier (ALA Scientific, Westbury, NY), and placed adjacent to a cell to detect oxidized CA transmitter. Recordings were collected from cells exposed to constant and uniform perfusion (~1 ml/min). Cells were perfused for 5-10 min with Hank's Buffered Saline Solution (HBSS). Basal CA release was measured during the HBSS perfusion for ~20 sec prior to changing to a high (50 mM) K^+ ^buffered saline solution containing in mM: 50 KCl, 87 NaCl, 1 MgCl_2_, 5 CaCl_2, _10 D-glucose, and 12 HEPES (pH = 7.3). Osmolarity of both HBSS and the 50 mM K^+ ^solutions were matched. Stimulated transmitter release was measured during the remainder of the 4 min recording period. All experiments were performed at ambient temperature, 22-24°C.

The amperometric signal was low-pass filtered at 2 kHz (8-pole Bessel, Warner Instruments, Hamden, CT). A 16-bit analog-to-digital converter (National Instruments, Austin, TX) was interfaced with custom-written software (graciously provided by Dr. Aaron P. Fox, University of Chicago, Chicago, IL), acquired at 10 kHz and stored on a personal computer. Root mean square (rms) noise was typically less than 1 pA. Amperometric spike features such as amplitude, quantal charge, and kinetic parameters were analyzed by custom-written analysis software (graciously provided by Dr. Eugene Mosharov of Columbia University, New York City, NY). The detection threshold for an event was set for 5 times the baseline rms noise level, and no trace was analyzed with rms noise greater than 2.5 pA. Overlapping events, those whose spike had not returned to baseline prior to the next event, were not considered in the analysis even though they were uncommon events. Rise time was measured over 25-75% of the spike's maximal amplitude. The area under individual amperometric spikes was measured as the charge (Q measured in pC) per release event.

Statistical analysis of the spike events and kinetic parameters was performed using the method described by Colliver *et al*. [58]. To determine whether there were any cells in a group that contained extreme values, a 'box and whiskers' plot was generated using the average peak amplitude, standard error and standard deviation from each cell in a group. Any cell with a mean value that was not within the same range as the majority of cells in a group was not considered for further analysis. For the statistical analysis of each kinetic parameter, the mean values for individual cells were used to generate the average value for the group of cells. This is referred to as the 'double mean' and was used to determine statistical significance by a t-test with unequal variance.

### Catecholamine analysis

CAs were identified and quantified by HPLC-EC as previously described [33,59,60]. Basal CA released was collected from cells exposed to HBSS. Stimulated release of CAs was collected from cells exposed to a stimulating solution of 50 mM K^+^. The samples were analyzed by a system that consisted of a Varian Pro-Star solvent delivery system and a model 9090 autosampler (Varian) coupled to a C18 column and an ESA Coulochem II detector. Separations were performed isocratically using a filtered and degassed mobile phase consisting of 12% methanol, 0.1 M sodium phosphate, 0.2 mM sodium octyl sulfate and 0.1 mM EDTA, adjusted to pH 2.7 with phosphoric acid. A computer was used to collect and store the chromatograms that were analyzed with Varian Star software.

### NPY analysis

Cells and samples were prepared and treated similarly for basal and stimulated release of NPY as for CAs except cells were stimulated for 15 min to evoke measurable NPY release, as previously determined for the optimal time course for stimulated NPY release [61]. NPY in the samples was purified with C18 Sep-Pak columns, (Peninsula Labs; San Carlos, CA) measured by an enzyme immunoassay and compared to a standard curve. The 96-well plate was read by a Powerwave × plate reader (Biotek instruments; Winooski, VT) and the calculation of sample value was analyzed by KC Junior Software (Biotek instruments). Basal and stimulated NPY release were normalized to total protein content determined by a BCA protein concentration assay (Pierce, Rockford, IL).

### Ca^2+ ^imaging

Intracellular Ca^2+ ^concentrations ([Ca^2+^]_i_) were measured using ratiometric fluorescence imaging with fura-2 (InCytIM2, Intracellular Imaging, Cincinnati, OH). Cells were replated 24 h before experimentation, loaded with fura-2, and prepared for recording as previously described [33,62]. Cells were washed for ~10 min prior to experimentation with HBSS while the cells were individually selected with imaging software. On each coverslip, 20-40 cells were individually imaged. Image pairs were obtained every 10 seconds at 340 nm and 380 nm wavelength. Backgrounds were subtracted from each wavelength, and the 340 nm image was divided by the 380 nm image to provide a ratiometric image. Ratios were converted to free [Ca^2+^]_i _by comparing data to fura-2 calibration curves made in vitro by adding fura-2 (50 μM free acid) to solutions that contained known [Ca^2+^] (0 nM to 602 nM; Molecular Probes-Invitrogen, Carlsbad, CA). Average resting and stimulated [Ca^2+^]_i _were determined from numerous independent experiments. Resting [Ca^2+^]_i _was measured during the first 180 sec of recording prior to stimulation with a high K^+ ^solution. Stimulated [Ca^2+^]_i _was calculated from the five points during the peak change in Ca^2+ ^levels that occurred in response to stimulation.

### Electron Microscopy

Cells that were untreated or forskolin-treated for 4 hrs were pelleted by centrifugation and fixed with ice-cold 3.5% glutaraldehyde in 0.1 mol/L sodium cacodylate buffer (pH 7.25) containing 5% sucrose and 2 mmol/L calcium chloride for 16 hours at 4°C. Cell pellets were washed in 0.1 mol/L sodium cacodylate buffer containing 5% sucrose (this and all subsequent steps up to polymerization were at room temperature) and postfixed in 1% osmium tetroxide in 0.1 mol/L sodium cacodylate buffer containing 5% sucrose for 3 hours. Cell pellets were then washed twice in distilled water, incubated for 1 hour in 2% aqueous uranyl acetate, dehydrated through graded ethanols to 100%, rinsed twice in propylene oxide, and infiltrated with a 1:1 mixture of Polybed resin (Polysciences, Inc., Warrington, PA) and propylene oxide for 3 hours. The cell pellets were then incubated in fresh Polybed resin for 3 hours, transferred to BEEM capsules filled with fresh resin, and polymerized overnight at 70°C. Ultrathin sections (0.05 μm) were cut with a diamond knife using a Reichert Ultracut E ultramicrotome (Depew, NY), collected on 200 mesh copper grids, post stained with uranyl acetate and lead citrate, and viewed and photographed with a JEOL (Peabody, MA) 100CX transmission electron microscope.

### Statistical analysis

All data are displayed as mean ± SEM. Statistical significance was determined by a t-test with unequal variance.

## Abbreviations

(SYT): synaptotagmin, (CA): catecholamine, (NPY): neuropeptide Y, (LDCV): large dense core vesicle, (FSK): forskolin, (HBSS): Hank's buffered saline solution, (HPLC-EC): high performance liquid chromatography-electrochemical detection.

## Authors' contributions

JMM helped conceive the experiment, performed all of the immunoblot experiments, most of the amperometry, NPY, immunocytochemistry, and Ca^2+ ^imaging experiments and assisted with the cell culture, DNA preparation and drafted the manuscript. JBP prepared most of the cells for experimentation, transfections, all of the HPLC, some of the immunocytochemistry and NPY experiments, and most of the plasmid DNA preparation. ABH helped to conceive the experiment, performed some of the cell culture, transfection, amperometry, and Ca^2+ ^imaging experiments, and prepared the final figures and manuscript. All authors have read and approved the final manuscript.
